# Continuous Estimation of Knee Joint Angle Based on Surface Electromyography Using a Long Short-Term Memory Neural Network and Time-Advanced Feature

**DOI:** 10.3390/s20174966

**Published:** 2020-09-02

**Authors:** Xunju Ma, Yali Liu, Qiuzhi Song, Can Wang

**Affiliations:** 1 School of Mechatronical Engineering, Beijing Institute of Technology, Beijing 100081, China; 3120195134@bit.edu.cn (X.M.); qzhsong@bit.edu.cn (Q.S.); 2 Shenzhen Institutes of Advanced Technology, Chinese Academy of Sciences, Shenzhen 518000, China; can.wang@siat.ac.cn

**Keywords:** sEMG, LSTM, time-advance, feature, estimation

## Abstract

Continuous joint angle estimation based on a surface electromyography (sEMG) signal can be used to improve the man-machine coordination performance of the exoskeleton. In this study, we proposed a time-advanced feature and utilized long short-term memory (LSTM) with a root mean square (RMS) feature and its time-advanced feature (RMSTAF; collectively referred to as RRTAF) of sEMG to estimate the knee joint angle. To evaluate the effect of joint angle estimation, we used root mean square error (RMSE) and cross-correlation coefficient *ρ* between the estimated angle and actual angle. We also compared three methods (i.e., LSTM using RMS, BPNN (back propagation neural network) using RRTAF, and BPNN using RMS) with LSTM using RRTAF to highlight its good performance. Five healthy subjects participated in the experiment and their eight muscle (i.e., rectus femoris (RF), biceps femoris (BF), semitendinosus (ST), gracilis (GC), semimembranosus (SM), sartorius (SR), medial gastrocnemius (MG), and tibialis anterior (TA)) sEMG signals were taken as algorithm inputs. Moreover, the knee joint angles were used as target values. The experimental results showed that, compared with LSTM using RMS, BPNN using RRTAF, and BPNN using RMS, the average RMSE values of LSTM using RRTAF were respectively reduced by 8.57%, 46.62%, and 68.69%, whereas the average *ρ* values were respectively increased by 0.31%, 4.15%, and 18.35%. The results demonstrated that LSTM using RRTAF, which contained the time-advanced feature, had better performance for estimating the knee joint motion.

## 1. Introduction

In recent years, the exoskeleton robot has attracted the attention of researchers from all over the world due to its broad application prospects in the fields of power assistance, disability assistance, and rehabilitation [1,2,3,4,5]. Human-machine cooperation technology can effectively improve exoskeleton comfort, which has become a current research focus in the exoskeleton field [4,6].

Electromyography (EMG) is a neuronal pulse control signal generated by the human central nervous system that reflects the contraction of related muscles. Electrodes attached on the surface of muscles can collect on the surface of EMG (sEMG) signals, retaining most of the information. In the case of non-invasive acquisition, sEMG has better stability and stronger anti-interference performance than EEG, occurring about 20–200 ms earlier than the corresponding muscle contraction [7,8]. There is a certain degree of delay when physical signals such as angle and angular velocity are used to estimate human motion intention. Therefore, sEMG is best used to estimate movement intention and as a control signal to realize human-machine cooperation in an exoskeleton.

sEMG signals are often used for action classification [9,10], torque estimation [11,12], and angle estimation. The joint angle is easy to measure and continuous joint angle estimation can help achieve smooth motion exoskeleton control and thus angle estimation has broad application prospects. The estimation process of the human joint angle based on surface EMG signals generally consists of the following steps: (1) signal acquisition, (2) signal processing, (3) feature extraction, and (4) model selection. The present research focuses on feature extraction and model selection. Optional features can be divided into time-domain features, frequency-domain features, and time-frequency-domain features, among which time-domain features are most commonly used [13,14,15].

Joint angle estimation methods based on sEMG can be generally divided into two categories. The first kind is based on the Hill muscular model [16,17]. As a biomechanical model, the Hill muscular model can establish the relationship between sEMG and the human joint angle. However, there are many parameters in the biomechanical model which must be determined and parameters are determined through experience. Because the muscle contraction changes drastically during human exercise, fixed model parameters may not effectively reflect continuous muscle movement. Therefore, the performance of the Hill muscular model when estimating joint angle estimation could be inferior.

In recent years, more and more researchers have used machine learning methods (the other kind of angle estimation methods) to estimate the joint motion angle based on sEMG. For example, support vector machines (SVM) [18], random forests (RF) [19], and other artificial neural network (ANN) algorithms [20,21,22,23] are often utilized to predict joint angles. SVM can be used for classification and regression. Li et al. [18] proposed a least squares support vector regression (LS-SVR) algorithm to estimate the joint angle in a human’s lower limb using multi-channel sEMG signals. LS-SVR is an improved version of SVM; it contributes to solving high computational SVM cost problems. sEMG integral absolute value (IAV) features seven lower limb muscles as LS-SVR inputs. Xiao et al. [19] utilized RFs with multiple time-delay features (five time-domain sEMG features and their time-delay features) to estimate the joint angle of the human lower limb. Zhang et al. [20] built an angle prediction model using a back propagation (BP) neural network with the RMS feature of sEMG to describe the relationship between the human leg joint angle and relevant muscle (sEMG) signals. In this study, four people with spinal cord injuries (SCI) and six able-bodied subjects participated in the experiment to verify the BPNN algorithm. The results showed that BPNN could achieve the continuous joint angle estimation. Chen et al. [21] used a deep belief network (DBN) to reduce multi-channel sEMG signal dimensions in order to reduce redundant information and extract effective information. Combined with the BP neural network, this further improved prediction accuracy. Arthur et al. [22] predicted shoulder and elbow joint motions using a time-delayed artificial neural network (TDANN) based on six shoulder and elbow muscles. Six able bodied subjects and two people with C5 tetraplegia participated in the experiment. Fei Wang et al. [23] used a general regression neural network adjusted with a general algorithm (GA-GRNN) and sEMG RMS feature to predict the knee joint angle. Shengxin Wang et al. [24] utilized a radial basis function neural network (RBF) as the joint angle model and extracted the liner profile-curve of sEMG as the input feature to estimate the human joint angle or angular velocity. Junhong Wang et al. [25] proposed a correlation dimension of a wavelet coefficient (WCCD) method to extract optimal feature vectors from five relevant muscles and used the Elman network to map the extracted sEMG features to knee joint angles. The above methods are all traditional machine learning methods.

With the development of deep learning technology, more deep learning algorithms have been proposed and used. Yongchuang Huang [26] used a recurrent neural network (RNN) to estimate the knee joint angle in real time and got desired results. RNN is a type of recurrent neural network that takes sequence data as an input, performs recursion in the evolution direction of the sequence, and connects nodes using a chain. The core task of RNN is to minimize the loss function of backpropagation to adjust parameters. When transfer time step numbers increase, the gradient of RNN shows an exponential decline trend until the gradient disappears. In order to solve the problem of RNN gradients disappearing, LSTM introduced self-loops to generate paths where the gradient flows for long continuance, which was invented based on RNN by (Hochreiter and Schmidhuber) [27]. Chao Wang [28] and Yan Chen [8] used LSTM to predict finger and upper limb joint angles.

However, the previous results are limited for the following reasons. Firstly, some of these studies did not consider that sEMG was generated earlier than the related muscle contraction, so various features adopted in some of the above methods could not readily reflect the real joint angle information. In order to solve this problem, we proposed a time-advanced feature of sEMG signals. In this paper, we selected a root mean square (RMS) feature and its time-advanced feature (RMSTAF; collectively referred to as RRTAF) of sEMG as the input feature of the joint angle estimation model. To the best of our knowledge, in the study of knee joint angle prediction based on sEMG, few people have adopted time-advanced features. Secondly, many previous studies did not use an algorithm suitable for sEMG sequencing. Considering that LSTM is suitable for time series data, we used LSTM to construct an angle prediction model. According to the literature, few people use LSTM to predict the human lower limb joint angle based on sEMG. Therefore, in this paper, we proposed a mothed of LSTM using RRTAF to predict the knee joint angle based on sEMG.

The remainder of this article is organized as follows. Section 2 presents the experimental and methodology, containing data acquisition, data processing, sEMG feature extraction, evaluation criteria of angle prediction effect, LSTM neural network for angle estimation, and BP neural network. Section 3 introduces the experimental results. Section 4 discusses the results and methods. Finally, Section 5 concludes the paper.

## 2. Experimental and Method

### 2.1. Data Acquisition

Five healthy subjects participated in this study (male, height: 177.30 ± 6.03, weight: 62.00 ± 4.65, foot length: 27.00 ± 0.55, BMI: 19.70 ± 0.47). The subjects’ physiological information is shown in Table 1. Subjects were asked to walk on a Bertec treadmill (Bertec Corporation, Columbus, OH, USA) (with an inclination angle of 0°) at a speed of 1.0 m/s for 3 min and then rest for 5 min between each walk. We chose one normal data set from three experiments for the method verification. All subjects were provided with an informed consent form for the experiment. The experiment was approved by the Ethics Committee at the Beijing Institute of Technology (Approval code: 2019007H).

**Figure 1:**
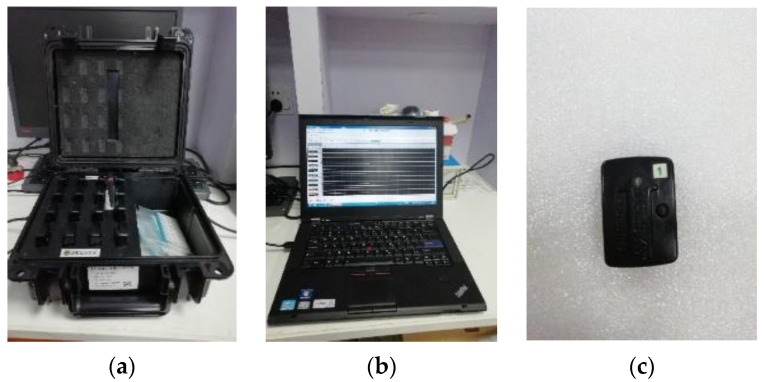
sEMG collection device, (**a**) TrignoTM Wireless EMG System; (**b**) operation community of Delsys software; (**c**) sEMG sensor.

**Figure 2:**
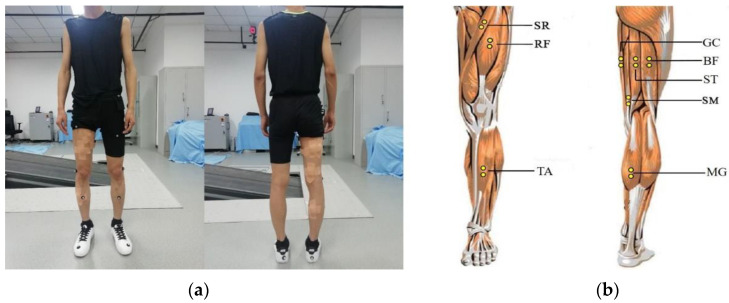
(**a**) Location diagram of sEMG sensors; (**b**) schematic diagram of human lower limb muscles used in this study.

The knee joint angles and related muscle sEMG signals of subjects’ right leg were simultaneously collected during walking. The muscles in the right leg, including the rectus femoris (RF), biceps femoris (BF), semitendinosus (ST), gracilis (GC), semimembranosus (SM), sartorius (SR), medial gastrocnemius (MG), and tibialis anterior (TA), were recorded using Delsys’s TrignoTM (Delsys Corporation, Natick, MA, USA) wireless EMG system (shown in Figure 1a–c). The sampling rate was 1200 Hz. The schematic diagram of the above muscles and the relevant sEMG sensors location diagram are shown in Figure 2. sEMG signal sensors were fixed with double-sided tapes and bandages. Before sEMG signal acquisition, to reduce the impedance between the sEMG sensor and skin, the relevant skin surface was wiped with alcohol to remove grease, and the hair on the skin surface was scraped off [20]. When collecting data, other electronic equipment in the laboratory were turned off or placed as far as possible from the experiment to reduce power frequency interference.

**Figure 3:**
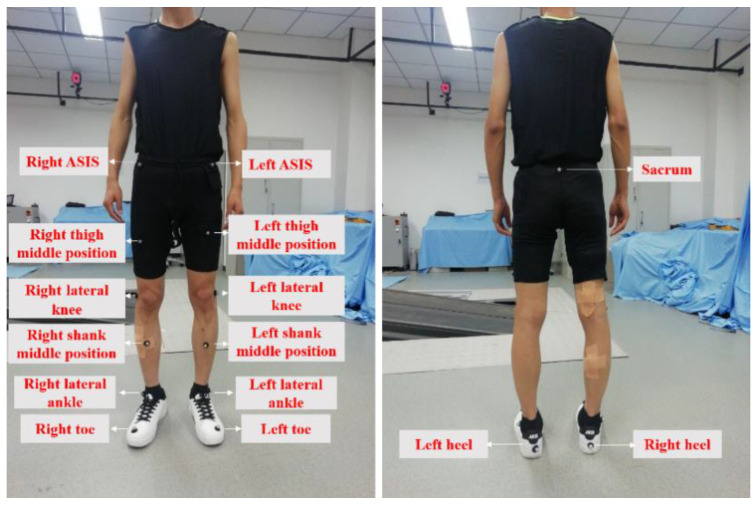
Location diagram of markers.

The knee joint angle of the subjects’ leg was obtained through a motion capture system with 12 cameras produced by the Motion Analysis Corporation (USA). The sampling frequency of the motion capture system was set as 120 Hz. The position and names of 15 markers when the subject was working are shown in Figure 3 and Table 2. This motion capture system integrated Delsys device so that the sEMG signal and kinematics data could be collected simultaneously.

### 2.2. Data Processing and Feature Extraction of sEMG

#### 2.2.1. Signal Preprocessing

The frequency of industrial power in China is 50 Hz and signal acquisition equipment is susceptible to industrial frequency interference during data acquisition. Therefore, the original signal is first subjected to a 50 Hz power frequency notch. The bandwidth of the EMG signal is 0.5–2000 Hz and the main energy is concentrated in 5–500 Hz [29,30]. In summary, the raw sEMG signals were processed by a notch filter with 50 Hz and a fourth-order Butterworth band-pass filter [31] with a bandwidth of 10–500 Hz to eliminate noise signals. The knee joint angle data was obtained using the OrthoTrack software (Motion Analysis Corporation, Santa Rosa, CA, USA) in the motion analysis system.

#### 2.2.2. Feature Extraction of sEMG and Time-Advanced Feature Signals

**Figure 4:**
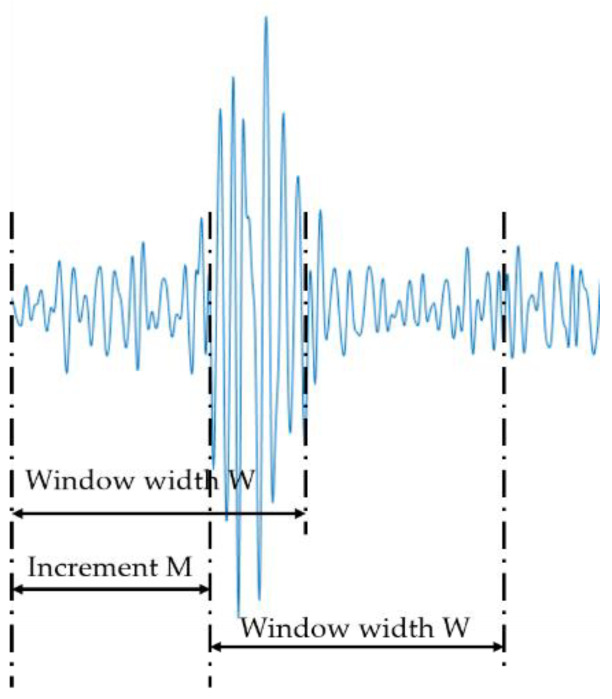
Schematic diagram of sEMG signals segmentation.

The features of an sEMG signal can be divided into three types: the time domain feature, frequency domain feature, and time-frequency domain feature. The time domain feature can be quickly and easily extracted without any transformation. The root mean square (RMS) value reflects the change of the amplitude of the sEMG signal voltage in the time dimension and variation of relevant muscle strength. In this study, we selected RMS as the sEMG feature, which was a time domain feature. The RMS feature of sEMG was obtained using a sliding window function in which a continuous split window was selected on the preprocessed sEMG signals and the width of the window was set to 20. The continuous split window method implies that the window width, W, and increment, M, are equal, which were all 20 in this paper. The schematic diagram of sEMG signals segmentation is shown in Figure 4.

The generation of sEMG signals was 20–200 ms earlier than the corresponding muscle action [7,8]. sEMG and angle data were collected at the same time. The angle data at this time corresponded to the sEMG at the previous moment. Therefore, in this paper we proposed a time-advanced feature of sEMG signals.

We moved the original sEMG signal sequence and angle sequence backward, then the moved sequences were delayed relative to the raw sEMG signal and raw angle signal. Therefore, the raw sEMG signal sequence was regarded as a time-advanced sEMG signal sequence, which corresponded to the delayed angle signal sequence. Before presenting the RMS feature and its time-advanced feature formulas of sEMG, *l* and *k* were proposed, as shown in Formulas (1) and (2). *l* represents the number of points that the angle sequence moves backwards, whereas *k* represents the number of points that the sEMG sequence moves backwards.

(1) $$l=ceil(\Delta t\times f_{angle}),$$

(2) $$k=l\times \frac{f_{sEMG}}{f_{angle}},$$

where Δt represents the advance time and set at 0.2 (s) (Δt∈[0.02,0.2]). $f_{angle}$ and $f_{sEMG}$ represent the sampling frequency of sEMG and joint angle, respectively. $f_{angle}\leq f_{sEMG}$ represents “ceil” as the function that returns the smallest integer greater than or equal to the specified expression.

The RMS feature extraction and its time-advanced feature (RMSTAF) formulas are shown in Formulas (3) and (4), where *emg*(*i*) represents the preprocessed sample sequence, *N* represents the width of the sliding window, and is set at 20. The feature curves obtained by the above method vibrated acutely. In order to obtain smoothness, a second-order Butterworth low-pass filter with a cut-off frequency 5 Hz was applied to above sEMG features.

(3) $$RMS(j)=\sqrt{\frac{1}{N}\sum_{i=(j-1)N+k+1}^{jN+k}emg^{2}(i)},$$

(4) $$RMS(h)=\sqrt{\frac{1}{N}\sum_{i=(h-1)N+1}^{hN}emg^{2}(i)},$$

where RMS(j) and RMS(h) represent the RMS feature of sEMG and its time-advanced feature relative to the corresponding angle data.

The sampling frequency of raw sEMG was 1200 Hz. After feature extraction, the sampling frequency of sEMG became 60 Hz. However, the sampling frequency of the knee angle was 120 Hz. In order to make the sEMG data and angle data be the same sampling frequency, the angle data was sub-sampled using Formula (5).

We set the lagging angle sequence relative to the raw angle signals as the incipient knee angle signals and the lagging sEMG sequence relative to the raw sEMG signals as the incipient sEMG signals. Then, the raw sEMG signals were used as time-advanced signals. Formula (6) represents the calculation formula of the joint angle sequence moving backwards or lagging behind the raw sEMG sequence.

(5) $$Angle_{s}(j)=\frac{1}{2}\sum_{2(j-1)+1}^{2j}Angle(i),$$

(6) $$Angle_{s}(h)=\frac{1}{2}\sum_{2(h-1)+l+1}^{2h+l}Angle(i),$$

where Angle(i) is the angle signal series before subsampling; $Angle_{s}(j)$ is the angle signal sequence after subsampling; $Angle_{s}(h)$ represents the joint angle sequence moving backwards or lagging behind the sEMG sequence.

### 2.3. Data Normalization

Since the values of angle data and sEMG data were not on the same order of magnitude, to get the angle prediction model faster and better, the above sEMG feature values and angle values after subsampling was normalized using min-max normalization [32]. Therefore, Formula (7) was used to restrict the above data to the range of [−1,1]. The test set was normalized with the normalization parameters of the training set.

(7) $$y(i)=y_{\min}+(y_{\max}-y_{\min})\times \frac{x(i)-x_{\min}}{x_{\max}-x_{\min}},$$

where x(i) represents the signal sequence before being normalized. $x_{\max}$ and $x_{\min}$ are the maximum and minimum values of x(i), respectively. y(i) is the normalized signal sequence; $y_{\max}$ and $y_{\min}$ are the maximum and minimum values of y(i), respectively. In this paper, we set $y_{\max}=1$ and $y_{\min}=-1$.

### 2.4. Long Short-Term Memory Neural Network for Angle Estimation

#### 2.4.1. Basic LSTM Structure

**Figure 5:**
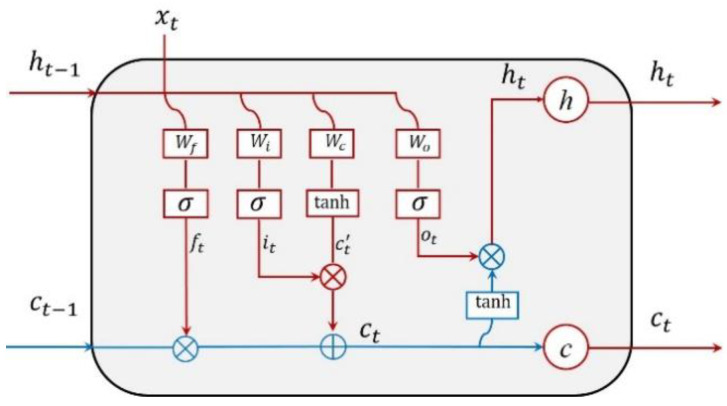
Complete structure of an LSTM memory block with one memory cell.

Hochreiter and Schmidhuber invented long short-term memory (LSTM) neural network on the basis of RNN [27]. Long time lags are unreachable to RNN architecture since the backpropagated error either decays exponentially or blows up [33]. LSTM introduced self-loops to generate paths where the gradient could flow, which thus solved the problem of the RNN gradient disappearing [27,34]. A hidden layer of LSTM contains a set of recurrently connected blocks, i.e., memory blocks. The input, output, and forget gates, as well as one or more recurrently connected memory cells consist in each memory block (Figure 5).

From Figure 5, $x_{t}$ represents the input data at time, *t*, or time step, *t*, in the sequence. $h_{t-1}$ and $h_{t}$ represent the value of the hidden layer at time, *t*, or time step, *t*, respectively. The *h* (calculated by Formula (13)) vector in the hidden layer contains the outputs of all LSTM cells. $f_{t}$ and $i_{t}$ respect the forget gate unit and input gate unit for time, *t*, or time step, *t*, respectively. Their values can be computed by Formulas (8) and (9). The values of the current input memory cell state, $c_{t}^{\prime }$, can be calculated by Formula (10). $c_{t}$ respects the memory information at time, *t*, or time step, *t*, which is updated from $c_{t-1}$ at time *t* − 1 or time step *t* − 1. Its values can be calculated by Formula (11). The values of the output gate unit $o_{t}$ was calculated by Formula (12).

(8) $$f_{t}=\sigma (W_{f}^{T}h_{t-1}+U_{f}^{T}x_{t}+b_{f}),$$

(9) $$i_{t}=\sigma (W_{i}^{T}h_{t-1}+U_{i}^{T}x_{t}+b_{i}),$$

(10) $$c_{t}^{\prime }=\tanh(W_{c}^{T}h_{t-1}+U_{c}^{T}x_{t}+b_{c}),$$

(11) $$c_{t}=f_{t}\circ c_{t-1}+i_{t}\circ c_{t}^{\prime },$$

(12) $$o_{t}=\sigma (W_{o}^{T}h_{t-1}+U_{o}^{T}x_{t}+b_{o}),$$

(13) $$h_{t}=o_{t}\circ \tanh(c_{t})$$

where σ(⋅) is sigmoid activation function and symbol ∘ represents that the corresponding elements are multiplied. $W_{f}^{T}$, $W_{i}^{T}$, $W_{c}^{T}$, and $W_{o}^{T}$, $U_{f}^{T}$, $U_{i}^{T}$, $U_{c}^{T}$, and $U_{o}^{T}$, and $b_{f}$, $b_{i}$, $b_{c}$, and $b_{0}$ are input weights, recurrent weights, and biases, respectively.

#### 2.4.2. Choice of LSTM Structural Parameters

**Figure 6:**
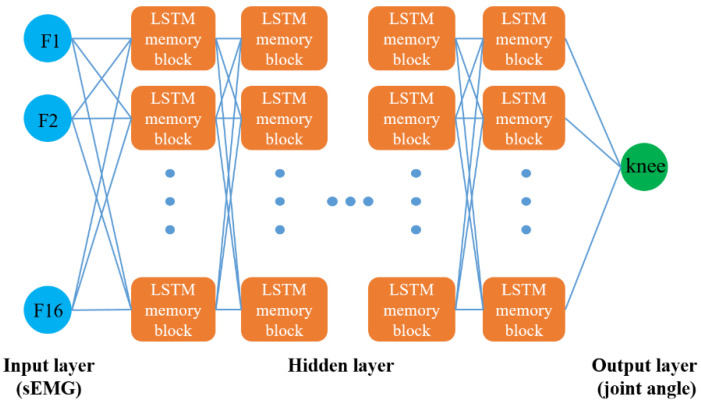
Topological structure of the LSTM network.

In this paper, we adapted a 100-layer LSTM network to train the knee angle prediction model, as shown in Figure 6. It consists of an input layer, many hidden layers, and an output layer. The input layer contains 16 nodes that are the RMS feature sequences and their time-advanced features of the above eight muscle sEMG signals after normalization. There are 200 hidden nodes in each hidden layer and one node in the output layer, which consists of knee joint angles. Then, the LSTM network can be trained by supervised learning and the target vector is the knee joint angle sequence collected simultaneously with the sEMG. The Adam optimization algorithm [35] with 200 rounds of training in a mini-batch size (50) was used to minimize the loss function and the learning rate was set to 0.0005. The dropout method was used as a regularization method and the drop probability was set to 0.5. The amount of data in the training set and test set accounted for 80% and 20% of the data set, respectively.

Estimating the joint angle is a regression problem. Therefore, we used the mean square error (MSE) to be the loss function, shown as Formula (14).

(14) $$cost=\frac{\sum_{i=1}^{n}{\left(\tilde{y_{i}}-y_{i}\right)}^{2}}{n},$$

where $\tilde{y_{i}}$ and $y_{i}$ represent the estimation angle and actual angle, respectively. *n* is the length value of the angle sequence.

### 2.5. BP Neural Network Algorithms

**Figure 7:**
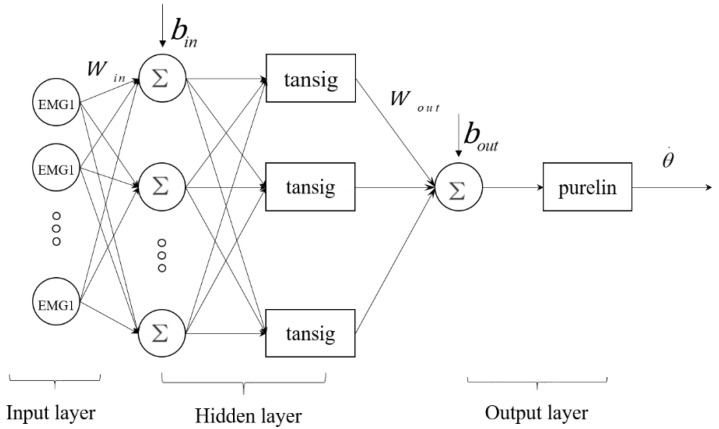
Structure of the BP neural network.

In this paper, we also selected a three-layer BP neural network (Figure 7) to construct the angle estimation model. The input and output vector were the same as the LSTM network. The tansig function and purelin function were used as the transfer functions from the input layer to the hidden layer, and from the hidden layer to the output layer [32]. The traingdm function was applied as the training function in the BP network. The learning goal, the learning rate, and the number of training epochs of the BP network were set to be 0.01, 0.1, and 50,000, respectively. The output angle of the BP network is shown in Equation (15).

(15) $$\dot{\theta }=\frac{1}{1+e^{-W_{out}[\frac{2}{1+e^{-2(W_{in}x+b_{in})}}-1]+b_{out}}},$$

where $\dot{\theta }$ is the predicted angle value, $W_{in}$ is the weight matrix of hidden layer, $W_{out}$ is the weight matrix of output layer, and $b_{in}$ and $b_{out}$ are the threshold vectors that correspond to the hidden layer and the output layer.

The range of optimal hidden-layer unit numbers was confirmed using the empirical Formulas (16)–(18). Then, the BPNN model was built with the above hidden layer units, computing the mean square error (MSE) (as shown in Formula (19)) between the estimated angle and actual angle. The number of hidden layer units corresponding to the minimum MSE value is the optimal number of hidden layer units. In this study, the optimal hidden layer unit number was 18.

(16) $$\overset{n}{\underset{i=0}{\sum }}C_{n_{1}}^{i}>k,$$

where k is the sample number, $n_{1}$ is the hidden unit number, and n is the input unit number. If $i>n_{1}$, then $C_{n1}^{i}=0$.

(17) $$n_{1}=\sqrt{n+m}+a,$$

where *m* is the output unit number, *n* is the input unit number, and *a* is a constant between [1,10].

(18) $$n_{1}=\log_{2}n,$$

where *n* is the input unit number.

(19) $$MSE=\frac{\sum_{i=1}^{n}{\left({\dot{\theta }}_{i}-\theta_{i}\right)}^{2}}{n},$$

where ${\dot{\theta }}_{i}$ is the estimated angle value, $\theta_{i}$ is the actual measured value of the angle, and *n* is the number of sampling points of the training sample.

### 2.6. Evaluation Criteria of Angle Prediction Effect

In this study, root mean square error (RMSE) and cross-correlation coefficient ρ were used to evaluate the effect of joint angle prediction. Their Formulas are shown in (20) and (21), respectively.

(20) $$RMSE=\sqrt{\frac{\sum_{i=1}^{n}\left({\dot{\theta }}_{i}-\theta_{i}\right)}{n}},$$

(21) $$\rho =\frac{\frac{1}{n}\sum_{i=1}^{n}\left(\theta_{i}-\bar{\theta })({\dot{\theta }}_{i}-\bar{\dot{\theta }}\right)}{\sqrt{\frac{1}{n}{\sum_{i=1}^{n}\left(\theta_{i}-\bar{\theta }\right)}^{2}}\sqrt{\frac{1}{n}{\sum_{i=1}^{n}\left({\dot{\theta }}_{i}-\bar{\dot{\theta }}\right)}^{2}}},$$

where ${\dot{\theta }}_{i}$ is the estimated angle value, $\theta_{i}$ is the actual measured value of the angle, *n* is the number of sampling points of the test sample, and $\bar{\dot{\theta }}$ and $\bar{\theta }$ are the average value of the estimated angle and the actual measured angle, respectively.

## 3. Results

**Figure 8:**
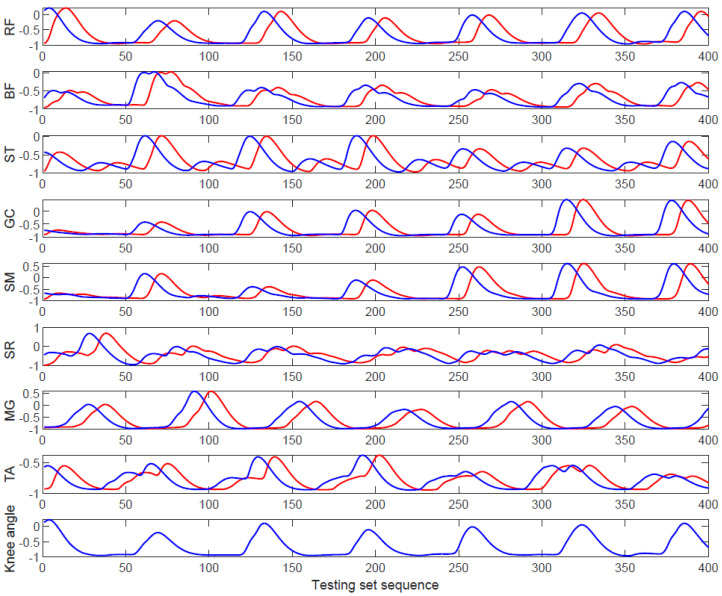
Curve of RRTAF (RMS and its time-advanced feature) of sEMG and curve of right knee joint angle after the normalization of subject 2. The blue line represents the RMS feature and the corresponding knee angle; the red line represents the time-advanced feature of RMS.

We verified whether RMS feature application and its time-advanced feature (RMSTAF) could effectively improve knee joint angle prediction accuracy. The method used RMS and RMSTAF feature signals of the eight muscles (sEMG) and the relevant knee angle as the input data and output data of the machine learning algorithms (LSTM neural network, BP neural network), respectively, to train the joint angle prediction model. The data curve of the input sequence and target sequence of LSTM is shown in Figure 8. The blue lines represent the RMS feature and knee angle; the red line represents the RMSTAF. Figure 8 shows the interrelationships among the RMS feature sequences, time-advanced feature, and joint angles. The angle data at this time corresponds to the sEMG at the previous moment. To prove the effectiveness of the time-advanced feature, the input data set was changed to the RMS feature of the sEMG signals of the above eight muscles. The BPNN algorithm was utilized to highlight the advantages of the LSTM algorithm in predicting the joint angle based on sEMG signals. In this paper, the above methods were named LSTM using RMS, LSTM using RRTAF, BPNN using RMS, and BPNN using RRTAF. By comparing the angle prediction results, we studied whether the application of the RMSTAF feature could improve knee joint angle prediction. The algorithm implementation and data processing software used in this study was MATLAB R2019a and the CPU of computer was Intel Core i5-10210U 2.11 GHz.

**Figure 9:**
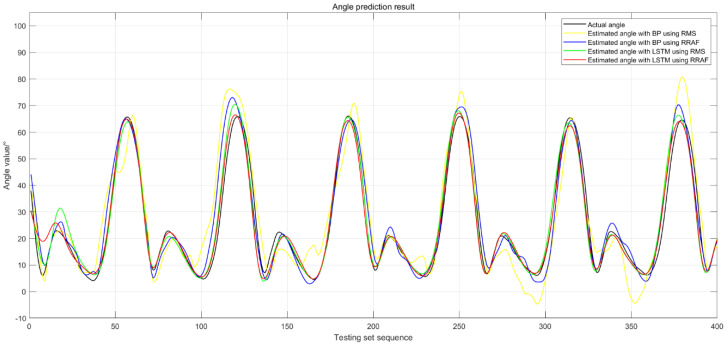
Knee angle estimation curves obtained with the four algorithms based on the data collected from subject 2 at a walking speed of 1.0 m/s. The black line represents the actual angle curve; the yellow, blue, green, and red lines represent the estimated angle with BPNN using RMS, BPNN using RRTAF, LSTM using RMS, and LSTM using RRTAF.

As an example, the knee angle estimation curves obtained via the above algorithms, which were based on data from subject 2 at a walking speed of 1.0 m/s, is shown in Figure 9. The black line represents the actual angle curve, whereas the yellow, blue, green, and red lines represent the estimated angle with BPNN using RMS, BPNN using RRTAF, LSTM using RMS, and LSTM using RRTAF, respectively. As can be seen from the figure above, the degree of similarity between the estimation curve and the actual curve is ranked as BPNN using RMS, BPNN using RRTAF, LSTM using RMS, and LSTM using RRTAF, from small to large. The estimation curve of BPNN using RRTAF, LSTM using RMS, and LSTM using RRTAF all have good smoothness. The smoothness of the curve obtained by BPNN using RMS is poorer than the above prediction curves. According to the comparison results, it can be concluded that LSTM using RRTAF had better performance in estimating the knee joint angle based on sEMG.

Table 3 shows each subject’s RMSE obtained with the four methods and their average values. For the five subjects, the RMSE values obtained by LSTM using RRTAF were within 2.6164–4.1744°, with an average of 3.4726 ± 0.6162°. Moreover, the RMSE range of LSTM using RMS, BPNN using RRTAF, BPNN using RMS were respectively 2.9764–4.6067° (average 3.7981 ± 0.6584°), 5.5089–7.8514° (average 6.5048 ± 1.8886°), and 8.6631–14.5964° (average 11.0913 ± 2.2334°). LSTM using RRTAF versus LSTM using RMS, BPNN using RRTAF versus BPNN using RMS, LSTM using RRTAF versus BPNN using RRTAF, and LSTM using RMS versus BPNN using RMS, the average RMSE values were reduced by 8.57%, 41.35%, 46.62%, and 65.76%. The average RMSE values of LSTM using RRTAF were reduced by 8.57%, 46.62%, and 68.69%, compared with those of LSTM using RMS, BPNN using RRTAF, and BPNN using RMS. The maximum decreased percentage of the RMSE of LSTM using RRTAF was 12.10%, compared to the RMSE of LSTM using RMS. The maximum decreased percentage of the RMSE of BPNN using RRTAF was 46.90%, compared to the RMSE of BPNN using RMS. (The analysis of the experimental results of each subject is shown in Appendix A.) A one-way analysis of variance (ANOVA) with the F test was used to compare the results of the four methods. The significant level was set at *p* < 0.05 and not adjusted during analysis. After a statistical test of significance, there were statistically significant differences among the four groups of RMSE data in Table 3. Except for no significant difference between RMSE data of LSTM using RMS and RMSE data of LSTM using RRTAF, the other pairwise groups all had significant differences. 

**Figure 10:**
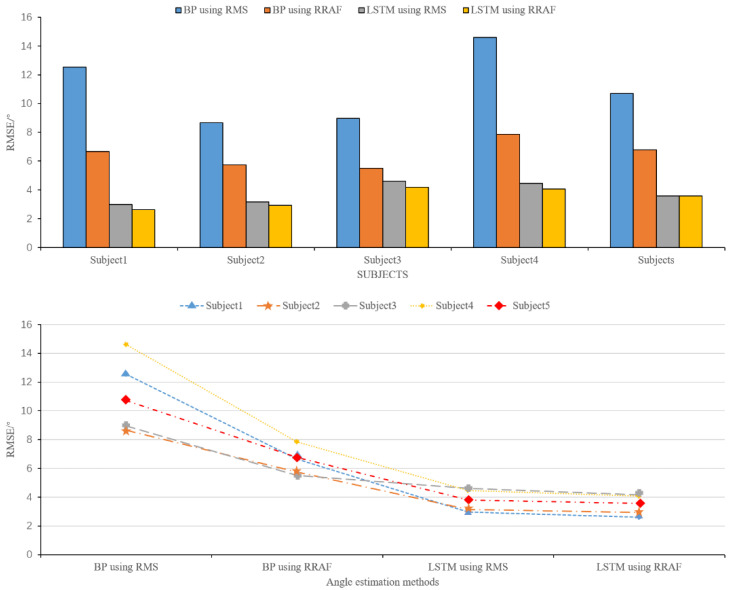
Comparison of the RMSEs between the estimated angle and the actual angle of knee joint obtained by the four methods (BPNN using RMS, BPNN using RRTAF, LSTM using RMS, LSTM using RRTAF) from subject 1 to subject 5.

Figure 10 shows the variation trend of the RMSE between the estimated angle and the actual angle of the knee joint obtained by the four methods (BPNN using RMS, BPNN using RRTAF, LSTM using RMS, LSTM using RRTAF) from subject 1 to subject 5. We can see that the RMSE obtained by LSTM using RRTAF was the smallest one for each subject, and the RMSE values obtained by BPNN using RMS, BPNN using RRTAF, LSTM using RMS, and LSTM using RRTAF gradually decreased for each subject.

The above results demonstrate that LSTM using RRTAF had better accuracy for estimating the knee joint angle. Adding a time-advanced feature can reduce knee angle estimation error. Further, LSTM had better accuracy than BPNN in predicting the knee joint angle based on sEMG.

Table 4 shows each subject’s cross-correlation coefficient *ρ* between the estimated angle and the real angle of the knee joint obtained using the four methods, as well as their average values. The *ρ* range of BPNN using RMS, BPNN using RRTAF, LSTM using RMS, and LSTM using RRTAF were 0.7682–0.9056 (average 0.8318 ± 0.0563), 0.9297–0.9625 (average 0.9451 ± 0.0139), 0.9732–0.9900 (average 0.9813 ± 0.0064), and 0.9788–0.9915 (average 0.9844 ± 0.0049). LSTM using RRTAF had the best performance of 0.9844 (SD = 0.0049). LSTM using RRTAF versus LSTM using RMS, BPNN using RRTAF versus BPNN using RMS, LSTM using RRTAF versus BPNN using RRTAF, and LSTM using RMS versus BPNN using RMS, the average *ρ* values increased by 0.31%, 13.62%, 4.15%, and 13.62%, respectively. The average *ρ* values of LSTM using RRTAF were reduced by 0.31%, 4.15%, and 18.35%, respectively, compared with LSTM using RMS, BPNN using RRTAF, and BPNN using RMS. The maximum increased percentage, *ρ*, of LSTM using RRTAF was 0.58%, compared to the *ρ* of LSTM using RMS. The maximum increased percentage, *ρ*, of BPNN using RRTAF was 20.46%, compared to the *ρ* of BPNN using RMS. (The analysis of the experimental results of each subject is shown in Appendix B) After the statistical test of significance, there were significant differences amongst the four *ρ* data groups in Table 4. Exept for having no significant difference between the *ρ* data of LSTM using RMS and *ρ* data of LSTM using RRTAF, the other pairwise groups all had significant differences. 

**Figure 11:**
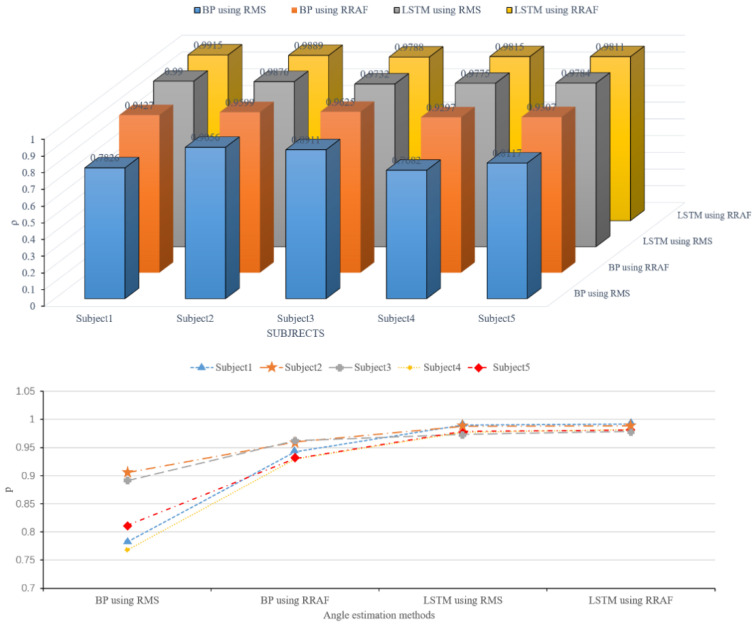
Comparison of the cross-correlation coefficient *ρ* between the estimated angle and the real angle of knee joint obtain by the four methods (BPNN using RMS, BPNN using RRTAF, LSTM using RMS, LSTM using RRTAF) from subject 1 to subject 5.

Figure 11 shows the variation trend, *ρ*, of the knee joint angle obtained by the four methods (BPNN using RMS, BPNN using RRTAF, LSTM using RMS, LSTM using RRTAF) from subject 1 to subject 5. The cross-correlation coefficient, *ρ*, obtained by BPNN using RMS, BPNN using RRTAF, LSTM using RMS, and LSTM using RRTAF gradually increased during each subject’s experiment. The *ρ* of LSTM using RRTAF was the biggest for each subject.

The above results indicated that LSTM was better than BPNN in terms of similarity between the predicted angle curve and the actual angle curve. Adding time-advanced features can further improve the similarity.

## 4. Discussion

This paper proposed a new continuous angle estimation method, i.e., LSTM using RRTAF (RMS and its time-advanced feature (RMSATF)) based on sEMG. The other three methods (i.e., LSTM using RMS, BPNN using RRTAF, and BPNN using RMS) were adopted to compare with LSTM using RRTAF. The results showed that LSTM using RRTAF had better performance in continuous knee joint angle estimation.

The RMSE values of BPNN using RRTAF compared to BPNN using RMS showed significant reductions. There was a statistically significant difference between the RMSE data of BPNN using RRTAF and the RMSE data of BPNN using RMS. Similarly, the cross-correlation coefficient *ρ* values of BPNN using RRTAF compared to these values of BPNN using RMS all had significant additions. There was a statistically significant difference between the *ρ* data of BPNN using RRTAF and the *ρ* data of BPNN using RMS. These results indicate that RRTAF of the sEMG contained more useful information than the single feature (RMS) in terms of angle prediction based on sEMG. Further, it effectively improved angle prediction accuracy.

The RMSE values of LSTM using RRTAF compared to these values of LSTM using RMS all had reductions. However, there was no statistically significant difference between the RMSE data of LSTM using RRTAF and the RMSE data of LSTM using RMS. Similarly, the cross-correlation coefficient *ρ* values of LSTM using RRTAF compared to these values of LSTM using RMS all had additions. There was no statistically significant difference between the *ρ* data of LSTM using RRTAF and the *ρ* data of LSTM using RMS. There may be three reasons why it is not statistically significant. One reason is that the RMSE and *ρ* of LSTM using RMS were both very small and very big. The second reason was that LSTM introduced self-loops to generate paths where the gradient flowed. In the LSTM structure, at time *t*, there were three inputs of the current neuron: the current input value $x_{t}$ at the current moment, $h_{t-1}$ at the previous moment, and the memory unit state $c_{t-1}$ at the previous moment. Each hidden LSTM layer not only contained the information of the current moment but was also part of the information of the previous moment (i.e., memory information). The RRTAF features of sEMG were composed of the RMS feature and its time-advanced feature. The advanced feature was as memory information during model training. The third reason was that the amount of evaluation criteria data was so small that a significance test was not conducted between the two data sets. However, the performance of LSTM using RRTAF results were better than LSTM using RMS for each subject’s experiment, showing that LSTM using RRTAF has the best application prospects in continuous knee angle predictions. Due to the differences between individuals and the third reason above, limited subjects participating in the experiment only permits a partial conclusion. Therefore, in the future, we will invite more subjects containing healthy men and women of various ages to participate in the experiment and improve the LSTM structure to better extract the information hidden in the time-advanced features. Moreover, extracting more effective sEMG features can be used as a further study in improving the accuracy of joint angle estimation.

From Figure 9, we can see that the similarity between the predicted curve and the actual angle curve at peaks and troughs was smaller than that at other places. This was because the vibration of muscles related to the movement of the human lower limbs at peaks and troughs was more intense. The similarity between the predicted curve obtained by LSTM using RRTAF and the actual angle curve at peaks and troughs was better than the other three methods. This result demonstrated that LSTM using RRTAF had a better learning ability than the other three methods. Due to individual differences, there were differences in the experimental results of the subjects. For example, the RMSE values of subject 3 and subject 4 obtained by LSTM using RRTAF are more bigger than the other three subjects. Therefore, in the future, there should be a focus on improving algorithms that can adapt to human differences.

## 5. Conclusions

sEMG occurs about 20–200 ms earlier than the corresponding muscle contraction. Based on this research result, we proposed a time-advanced feature to improve the angle estimation performance. We used a LSTM with RMS feature and its time-advanced feature of the sEMG to estimate knee joint angle for five healthy subjects. In order to highlight the good performance of the proposed method, three other methods (i.e., LSTM using RMS, BPNN using RRTAF, and BPNN using RMS) were compared with it. The experiment showed that the average RMSE and *ρ* between the estimated angle and real angle of LSTM using RRTAF were 3.4726 (SD = 0.6162) and 0.9844 (SD = 0.0049), respectively. Compared with LSTM using RMS, BPNN using RRTAF, and BPNN using RMS, the average RMSE values of LSTM using RRTAF were reduced by 8.57%, 46.62%, and 68.69%, respectively. Further, average *ρ* values were increased by 0.31%, 4.15%, and 18.35%, respectively. The average RMSE and *ρ* of BPNN using RRTAF were reduced by 41.35% and increased by 13.62%, when compared with BPNN using RMS. The results proved that the time-advanced feature improved the accuracy for estimating knee joint motion. LSTM was more suitable for knee joint angle estimation based on sEMG. The method proposed in this paper has wide potential application in human-machine smooth motion control and can effectively improve human-machine coordination performance in the exoskeleton.

In the future, we will invite more healthy men and women of various ages to obtain more samples, which will further verify our method proposed. Moreover, we will add a real-time feedback mechanism to improve the robustness of the algorithm and use the improved method to control the exoskeleton in real time. We also will focus on improving the algorithm to adapt to individual differences.

## Figures and Tables

**Table 1:** The subjects’ physiological information.

Subjects	Age	Height (cm)	Weight (kg)	Foot Length (cm)	Foot Width (cm)
1	23	168	55	26.5	10
2	25	174	58	26.5	10
3	25	178.5	65	27	10
4	28	186	67	28	10
5	22	180	65	27	10
Average	25	177.3	62	27	10

**Table 2:** Position names of the subjects’ 15 markers.

Number	Name	Number	Name
1	Right anterior superior iliac spine (ASIS)	9	Right toe
2	Left anterior superior iliac spine	10	Left thigh middle position
3	Sacrum	11	Left lateral knee
4	Right thigh middle position	12	Left shank middle position
5	Right lateral knee	13	Left lateral ankle
6	Right shank middle position	14	Left heel
7	Right lateral ankle	15	Right toe
8	Right heel		

**Table 3:** RMSE obtained by the four methods for each subject.

Subject	BPNN Using RMS	BPNN Using RRTAF	LSTM Using RMS	LSTM Using RRTAF
1	12.5307	6.6535	2.9764	2.6164
2	8.6631	5.7339	3.1535	2.9229
3	8.9609	5.5089	4.6067	4.1744
4	14.5964	7.8514	4.4482	4.0728
5	10.7052	6.7764	3.8055	3.5764
Average	11.0913 ± 2.2334 (*,$)	6.5048 ± 1.8886 (#,^)	3.7981 ± 0.6584 (&)	3.4726 ± 0.6162 (~)

*, $, #, ^, and ~ denote a statistically significant difference (*: *p* (between BPNN using RMS and BPNN using RRTAF) < 0.05; $: *p* (between BPNN using RMS and LSTM using RMS) < 0.05; #: *p* (between BPNN using RRTAF and LSTM using RMS) < 0.05; ^: *p* (between BPNN using RRTAF and LSTM using RRTAF) < 0.05; &: *p* (between LSTM using RMS and LSTM using RRTAF) > 0.05; ~: *p* (between BPNN using RMS and LSTM using RRTAF) < 0.05).

**Table 4:** Cross-correlation coefficient *ρ* obtained by the four methods for each subject.

Subject	BPNN Using RMS	BPNN Using RRTAF	LSTM Using RMS	LSTM Using RRTAF
1	0.7826	0.9427	0.9900	0.9915
2	0.9056	0.9599	0.9876	0.9889
3	0.8911	0.9625	0.9732	0.9788
4	0.7682	0.9297	0.9775	0.9815
5	0.8117	0.9307	0.9784	0.9811
Average	0.8318 ± 0.0563 (*,$)	0.9451 ± 0.0139 (#,^)	0.9813 ± 0.0064 (&)	0.9844 ± 0.0049 (~)

*, $, #, ^, and ~ denote statistically significant difference (*: *p* (between BPNN using RMS and BPNN using RRTAF) < 0.05; $: *p* (between BPNN using RMS and LSTM using RMS) < 0.05; #: *p* (between BPNN using RRTAF and LSTM using RMS) < 0.05; ^: *p* (between BPNN using RRTAF and LSTM using RRTAF) < 0.05; &: *p* (between LSTM using RMS and LSTM using RRTAF) > 0.05; ~: *p* (between BPNN using RMS and LSTM using RRTAF) < 0.05).

## Appendix A

**For each subject, the experiment results about RMSE is as follows:**

- From subject 1 to subject 5, the RMSE values of LSTM using RRTAF are respectively reduced by 12.10%, 7.31%, 9.38%, 8.44%, 6.02% and the maximum decreased percentage is 12.10% (subject1), compared to the RMSE values of LSTM using RMS.
- From subject 1 to subject 5, the RMSE values of BPNN using RRTAF are reduced by 46.90%, 33.81%, 38.52%, 46.21%, 36.70%, respectively, and the maximum decreased percentage is 46.90% (subject1), compared to the RMSE values of BPNN using RMS.
- From subject 1 to subject 5, the RMSE values of LSTM using RRTAF are reduced by 60.68%, 49.02%, 24.22%, 48.13%, 47.22%, respectively and the maximum decreased percentage is 60.68% (subject1), compared to BPNN using RRTAF.
- From subject 1 to subject 5, the RMSE values of LSTM using RMS are respectively reduced by 26.50%, 9.05%, 9.21%, 27.25%, 20.54%, respectively, and the maximum decreased percentage is 27.25% (subject4), compared to the RMSE values of BPNN using RMS.

## Appendix B

**For each subject, the experiment results about *ρ* is as follows:**

- Compared to BPNN using RMS, *ρ* of BPNN using RRTAF are respectively increased by 20.46% (subject1), 6.00% (subject2), 21.02% (subject3), 8.01% (subject4), 14.66% (subject5), the maximum added percentage is 21.02% (subject3) and the average value is increased by 13.62%.
- The *ρ* values of LSTM using RRTAF are respectively increased by 0.15% (subject1), 0.13% (subject2), 0.58% (subject3), 0.41% (subject4), 0.28% (subject5), the maximum added percentage is 0.58% (subject3) and the average value is increased by 0.31%, compared to LSTM using RMS.
- Compared to BPNN using RRTAF, *ρ* of LSTM using RRTAF are respectively increased by 5.41% (subject1), 3.02% (subject2), 1.70% (subject3), 5.28% (subject4), 5.42% (subject5), the maximum added percentage is 5.42% (subject5) and the average value is increased by 4.15%.
- Compared to BPNN using RMS, *ρ* of LSTM using RMS are respectively increased by 26.50% (subject1), 9.05% (subject2), 9.21% (subject3), 27.25% (subject4), 20.54% (subject5), the maximum added percentage is 27.25% (subject4) and the average value is increased by 13.62%.
